# A Meta-Agent Based Approach to Exploit the Collective Product of Mobile Cyber-Physical Collectives

**DOI:** 10.3389/frobt.2022.904819

**Published:** 2022-06-24

**Authors:** Afra Khenifar, Jean-Paul Jamont, Michel Occello, Choukri-Bey Ben-Yelles, Mouloud Koudil

**Affiliations:** ^1^ LMCS Laboratory, École Nationale Supérieure D’Informatique (ESI), Algiers, Algeria; ^2^ Institute of Engineering, University Grenoble Alpes, Valence, France

**Keywords:** multi-agent systems, collective product, exploitation of emergent, phenomenon, interoperability, Wireless Sensor Network, Unmanned Aerial Vehicle system

## Abstract

A cyber-physical system (CPS) is a system with integrated computational and physical abilities. Deriving the notion of cyber-physical collective (CPC) from a social view of CPS, we consider the nodes of a CPS as individuals (agents) that interact to overcome their limits in the collective. When CPC agents are able to move in their environment, the CPC is considered as a Mobile CPC (MCPC). The interactions of the agents give rise to the appearance of a phenomenon collectively generated by the agents of the CPC that we call a collective product. This phenomenon is not recorded as “a whole” in the CPC because an agent has only a partial view of its environment. This paper presents COPE (COllective Product Exploitation), an approach that allows one MCPC to exploit the collective product of another one. The approach is based on the deployment of meta-agents in both systems. A meta-agent is an agent that is external to a MCPC but is associated with one of its agents. Each meta-agent is able to monitor the agent with which it is associated and can fake its perceptions to influence its behavior. The meta-agents deployed in the system from which the collective product emerges provide indicators related to this product. Utilizing these indicators, the meta-agents deployed in the other system can act on the agents in order to adapt the global dynamics of the whole system. The proposed coupling approach is evaluated in a “fire detection and control” use case. It allows a system of UAVs to use the collective product of a network of sensors to monitor the fire.

## 1 Introduction

**Context** An agent is an entity immersed in an environment that it can perceive and act on. It is capable of making decisions and acting autonomously in the environment, in order to achieve the objectives assigned to it. It has reactive capabilities to deal with the dynamics and uncertainties of the changing state of its environment. However, it is not only event-driven. It is also proactive and, as such, it can initiate interactions with other agents. A multi-agent system (MAS) is a set of agents in a shared environment of which each agent has only a local perception. These agents are endowed with self-organizing capabilities that allow them to adaptively modify their organizational structure according to their environment. The local interactions between the agents of the MAS give rise to an emerging global phenomenon called “a collective product” whose complete representation is not known to the agents: it can only be observed by an external observer.

In this article the notion of cyber-physical collectives (CPC) comes from a social vision of cyber-physical systems (CPS). We consider the nodes of a CPS as individuals (agents) who cooperate to overcome their lack of knowledge and competence in order to achieve goals that they are not able to realize.

**Problem** Consider two MCPCs that were developed by different designers. They do not have the ability to interact because of their functional and technological heterogeneity: they act independently of each other. Assuming that the agents that make up the two MCPCs are black boxes because the source code is not available, the general addressed problem is “How do we get these independent MASs to interact so that the collective product of one can be exploited by the other?.

**Illustrative scenario** In the context of forest fire detection, a wooded area is monitored. A wireless instrumentation system has been deployed to measure physical parameters from the environment. Data are collected and processed by a Local Alerting Control Unit (LACU). When variations of the environmental parameters are interpreted as the sign of a fire, a fire department is then alerted. Firemen must move around the site to validate the presence of the fire and apply firefighting procedures.

The fire department wishes to be able to remotely confirm the suspicion of fire *via* a real-time video system. This video support must be maintained during fire fighting. The designer uses a multiple Unmanned Aerial Vehicles (UAV) surveillance system which proposes an in-flight visual monitoring of the neighborhood of its area of deployment.

*The wireless instrumentation system:* this system is a Wireless Sensor Network (WSN). It is a group of spatially distributed and dedicated autonomous sensor nodes for monitoring the physical conditions of the environment and the transmission of detected event to a central location.

Sensor nodes have three main functions:

• a measure function: nodes capture data from their physical environment.
• a transmission function: one node can communicate with neighbors within range.
• a decision function: nodes are able to process data and trigger actions.

The system is viewed as a MAS, where sensors nodes are seen as agents which interact together to:

• make the detection of fire more reliable.
• enable the transmission of risk alert messages to LACU by agents not directly connected to it (problem of limited emission ranges).

**FIGURE 1:**
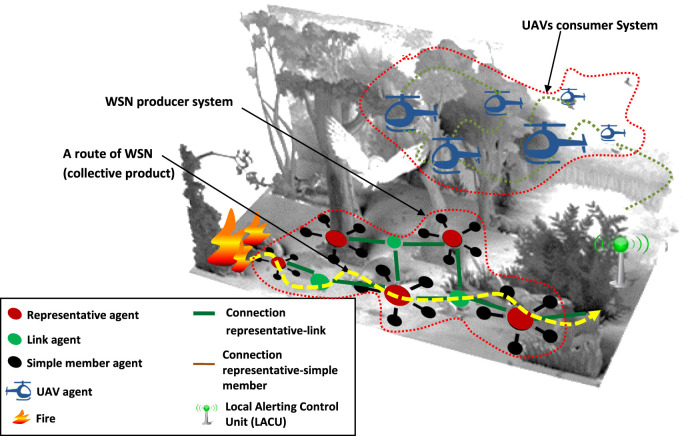
Illustrative use case.

Because the network coverage area is much larger than the emission radio range of a single node, agents use a multi-hop routing. A route is a sequence of directly connected agents forwarding messages from the source agent to the target. In order to search routes between a sensor and the LACU, the wireless instrumentation system uses the MWAC model (Jamont et al., 2010; Hamani et al., 2018). This model reduces the induced energy consumption by establishing an organization that limits the number of active connections during the route search process. For this purpose, MWAC defines three roles that can be assigned to the agents. A *representative* agent manages and routes the messages of agents that are directly connected to it. To achieve this task, it broadcasts, relays and responds to route search requests. A *link* agent enables message exchange between the representative agents that are directly connected to it. A *simple member* agent communicates only with the representative node to which it is directly connected. It does not have any routing task to ensure, unless it is the first sender or the last receiver of a message. Therefore, MWAC route is a succession of agents with the roles of *link* or *representative* (Figure 1). When an agent whose role is not “representative” decides to send a message i.e. it detects a temperature above a given threshold, it shall solicit its representative agent. To find the route, the representative agent sends route request messages and receives route reply messages including the route represented as a list containing only representative agent identifiers. Once the route is found, the representative agent sends a message of type MWACRoutedData. This message contains the temperature taken by the sensor agent (source of the information) and the MWAC route to follow. As it is often the case in bit efficient routing protocol, when the message is repeated by an agent, it contains only the remaining part of the route to follow.

A MWAC route is created to allow the transmission of the fire risk alert from an agent to the LACU. The agents do not have a complete representation of the route followed by the message^1^: the route is *the collective product* of the agents of this system. The observation of a route induces the following interpretation: a fire is suspected.

*The aerial video support system:* this system is composed of several interacting UAVs. It proposes an in-flight visual monitoring of the neighborhood of their areas of deployment.

Each UAV has three main functions:

• a photograph function: an UAV can take aerial photos.
• a transmission function: an UAV can communicate with neighbors within range or with an external entity (using two different communication protocols).
• a movement function: an UAV can move to a given position and remain in hover flight.

The UAV system (UAVS) is viewed as a MAS. UAVs are agents which interact together to:

• perform a flight formation: allows the UAVS to cover the desired area.
• enable the aerial picture transmission: each individual picture is communicated to the LACU as a part of the global picture.

Covering the area to be observed requires the UAVs to establish a mesh. Each UAV positions itself in relation to its neighbours. No single UAV agent maintains a complete representation of the mesh. The global mesh is *the collective product* of the UAVS. The mesh is formed to allow the LACU to create the overall aerial visual image of the monitored area by combining individual UAV images.

*The WSN collective product exploitation by the UAVS:* when a fire is suspected, firefighters subsequently deploy the UAVS to the area monitored by the WSN. They want the UAVs to move themselves to the area where the fire is supposed to be, without the sensor nodes being able to locate them (no global positioning system). UAV agents have to (Figure 1):

1) Fly up the route to the critical point.
2) Spread over the area surrounding the critical point.
3) Transmit the pictures to allow a decision making.

The last two steps match the functions offered natively by the UAVS. The first step requires guiding the UAVs to the fire without the GPS position of the critical point.

Sensors in the vicinity of the fire are supposed to periodically generate alert messages that travel the network using a route from the fire to the LACU. To realize the first step, the UAVS has to exploit the route i.e. the WSN^2^ collective product.

A transmitted frame always carries the address of its sending agent and the address of the destination neighboring agent (the datalink layer addresses) in addition to the MWAC routed message (the payload). Listening to these frames will allow an UAV agent to get closer to the frame sender using a Received Signal Strength Indication (RSSI) based algorithm. The route will be followed by the UAV in the opposite direction than the one taken by the message.

**Contribution** The main contribution of this article is the proposal of a new approach for coupling two MCPCs. This approach named COPE (COllective Product Exploitation) allows one MCPC to exploit the collective product of another MCPC. The approach is based on the deployment of meta-agents in both systems. A meta-agent is an agent that is external to a MCPC but is associated with one of its agents. Each meta-agent is able to monitor the agent with which it is associated and can fake its perceptions to influence its behavior. Another contribution is the definition and implementation of a meta-agent architecture based on the use of perception, decision and action rules.

**Organization of the article** This paper is structured as follows. In Section 2, the related work with the exploitation of collective products is presented. Section 3 provides an overview of the newly proposed COPE approach and presents the meta-agent architecture. Section 4 emphasizes the key elements for implementing and setting up the COPE approach in the context of the illustrative scenario. Section 5 provides an assessment of COPE implementation.

## 2 Related Work

We consider that a designer wants a multi-agent system, called a consumer MAS, to exploit the collective product of another multi-agent system called a producer MAS. We consider that the following steps are necessary for a designer to implement the exploitation of a collective product:

1) Using a model that describes the link between how the agents in the producer MAS operate and what they collectively produce. This model helps to understand how complex high-level behaviors are produced, starting from simple interactions between agents. In the following, we call such a model an *understanding model*.
2) Exploiting the understanding model with information available in the two MAS to enable the collective product to be detected.
3) Acting on the consumer MAS in order to allow it to exploit the collective product of the producer MAS.

### 2.1 Understanding the Articulation Between Local Behaviours and Collective Behaviour

Two complementary processes are often used to study the link between agent individual behavior and collective behavior. The first one consists in analyzing the available execution traces to identify global behavior patterns from the observations of low-level agent behaviors. The other process consists in reproducing in simulation the analyzed system in order to understand this link through iterative experiments.

**Analysis of execution traces** can be used when it is possible to record information about the evolution of the environment, the activity of the agents and their interactions by observing the system.

In many works in the literature, the simulation of the complex system that produces the emergent phenomenon is implemented. In order to collect data, event logs are a commonly used solution (Schecter et al., 2018; Bemthuis et al., 2019; Anastassacos et al., 2020). The nodes of the system here are not black boxes and the environment is completely accessible because it is simulated.

The problem is more difficult when working with a physically distributed system in which the environment and the nodes are not completely accessible (the nodes of the system are black boxes). In this case, it is necessary to instrument the system producing the emergent phenomenon to produce observables/traces. We have identified few works dealing with this problem, but one original work (Lamarche Perrin et al., 2014a; Lamarche-P et al., 2014b), that was used in the context of distributed simulation, deploys distributed software probes that collect specific data on the system components called microscopic information. These probes use distributed computing tasks to abstract the collected data into “facts”.

Once observables are available, the traces must be analyzed. Some solutions use algorithms for understanding the emergent behavior. At this end, in (Mirchevska et al., 2014) the MAS activity is represented as a graph where graph’s edges correspond to the performed actions and graph’s nodes correspond to the environment states at action starts. The paths in the graph with the highest number of merged actions allow for more traces to be analyzed, so these paths are labeled as emerging strategic behaviors. As an illustration of this type of approach, in (Ramos and Ayanegui, 2008; Aler et al., 2009) the training behavior of football agents during a game is studied. The formations are represented by planar topological graphs. The trajectory of the ball on the graph is studied in order to understand the different behaviours such as passing, dribbling, and attack support.

To study the causal relationships between micro and macro layers, Singh et al. (2017) propose a simulation framework where the system behavior data are collected from the simulation and imported into an ontology. This ontology provides support for both system-level and component-level behavior modeling. It distinguishes between internal (component) and external (inter-component) behaviors and aggregates the component behaviors and their interactions into a single model. A behavior classifier then automatically infers the type of behavior using the main class of the ontology named “emergent behavior”.

Serrano et al. (2009) propose an intelligent data analysis technique to understand the emergent behavior of a MAS using a three-phase process: a retrieval phase, a pre-processing phase and a data mining phase. The approach consists of analyzing and interpreting execution traces containing agent-specific information, inferring relationships between entities, making estimates of knowledge under construction and interpreting the total amount of data.

**Process focused on the reproduction of the phenomenon in simulation** adopts an experimental approach, whose principle is to “understand by reconstructing” the emergent phenomenon. To this end, it is proposed to assemble “puzzles” using a bottom-up approach. In (Mataric, 1993), a set of behavioral primitives is proposed in a mobile robotics context. These primitives are for example collision avoidance, following, dispersion, etc. In order to understand the collective behavior of a group of human beings, Will (2016) propose to define shared behavioral rules called norms. As an illustration, convergence is a collective behaviour where agents’ orientations are strongly aligned, agents’ positions are spaced apart and the resulting collective movement follows a linear trajectory, it then has a link with the norms of oneness and communication.

The epsilon machines have also been used to understand emergent phenomena, for example in supply chain networks (Surana et al., 2005). They work according to a top-down approach: an epsilon machine starts from observed macroscopic data and deduces the simplest causal structure, instead of starting from a microscopic description of the parts and their interactions to deduce macroscopic phenomena. The epsilon machine generates observations and describes the intrinsic computation of the system, i.e., the way it stores and processes information. Based on a probabilistic calculation of the reappearance of these observations in the future, it could be able to characterize the macroscopic phenomenon. The epsilon machines thus represent a special class of deterministic finite state automata whose transitions are labeled with conditional probabilities and which can thus also be considered as Markov chains (Surana et al., 2005).

From a social perspective, dynamical systems analysis can be used to develop computational models to show how simulations of individual decisions can create emergent organization within a group (Reed and Vallacher, 2020). The advantages of these models are: 1) the clarity and objectivity of the interpretations, 2) their ability to provide intermediate explanations which link individual behavior to group behavior, and 3) their use of real-world data. These models help to describe emergent behavior on multiple levels, without assigning priority to any particular level so as not to exclude interactions between the different levels. These approaches have been used to study emergent phenomena in several systems such as: ants (social insects foraging for food), traffic jams (automotive transportation system), slime molds (self-organizing unicellular organisms), segregation (human social system), and wolf-sheep predation (food web ecosystem).

### 2.2 Detecting Emerging Phenomena

Approaches to detect emergence are classified into three categories (O’Toole et al., 2014):

*Approaches based on variables* aim to characterize the global state of the system by using a set of observable variables. It is then possible to detect the occurrence of an emergent phenomenon when these variables present particular properties. They are used when system modeling details are known. An example of a variable-based approach is the use of system entropy as a measure to indicate emergence. For example, Mnif and Müller-Schloer (2011) argue that entropy decreases with the increase of order whereas emergence should increase with order thanks to self-organized processes between the elements of a system compared to a starting condition of maximum disorder. In their experiments, they use a chicken simulator, whose objective is to explain the behavior behind the injury of chickens crowded into cages to avoid a large loss of animals. In simulating this behavior, order patterns, representing entropy decrease, appear in the form of swarms of chickens. This is a case of “negative” emergence, i.e., unwanted, since the overall objective is to reduce the mortality rate of the chickens. A controller is then set up to perform actions aiming at dispersing the swarms and thus increasing the entropy.

*Formal approaches* attempt to identify and investigate the underlying causes of emergence in system design using simulation and formal language techniques. An example is the use of a formal grammar to define weak emergence (Kubí, 2003). In these approaches, emergence is defined as a set of properties generated by interactions between agents but which cannot be derived by summing up individual behaviors through a superposition of all individual agent languages on each other. These approaches are highly centralized and require that all possible agent behaviors are known and remain unchanged throughout the systems’ life cycle. They also require, at the design stage, a detailed modeling of the system using a formal language. For example, the authors Moshirpour et al. (2012) present a technique based on formal models to detect emergent behavior at the time of system design. At this stage, they aim to determine the cause of the behavior and try to control the emergent phenomena.

*Event-based approaches* are a hybridization of previous approaches. They are event-based and therefore require knowledge of *system modeling details* to specify the events to be detected, and at the same time they use events as parameters to detect emergence *at runtime*. An event-based approach is best suited to detect emergent phenomena in complex distributed adaptive systems (O’Toole et al., 2014; Liu et al., 2018). An example of an event-based approach is the solution of David et al. (2009), where events are called emergent facts, and their occurrence is detected by deploying software probes in the simulation tool. The probes act as an external observer of the MAS based on these facts. Assuming that emergence is knowledge at a higher level of description (David et al., 2009), the detection of facts during the simulation allows the emergent phenomenon to be represented as meta-knowledge. In (Chen et al., 2008), the authors begin by identifying two types of events to be detected according to the level of abstraction, either simple or complex events. Simple events are agent state transitions seen from a particular level of abstraction, and complex events are a particular configuration of simple events where the dimensions of time and space are included. Once all events are formally described, the statistical analysis and the understanding model are used to enable the identification of relationships between the levels of abstraction of the system that lead to the detection of emergence at runtime. An original work aims at the suppression of the effect of emergent phenomena interfering negatively with the objective of the system producing it (Liu et al., 2018). For this purpose, sensors are added during the construction of the system that can give rise to such a disturbance. The role of these sensors is to update a set of parameters (speed, position, etc.) as soon as an event is detected (movement of an UAV belonging to an adversary). Subsequently, they use thresholds to determine whether the system is in phase to detect the collective behaviour of a swarm of UAVs.

### 2.3 Adopted Guideline

To the best of our knowledge, there is no work focusing on exploiting the collective product of one MAS by another MAS (the third step). However, this work can be related to studies in which there are interactions between groups of individuals that can sometimes implicitly use emergent properties. For example, a team of two artificial dogs, driven by simple reactive controllers, is tasked with herding a flock of heterogeneous sheep (Bennett et al., 2021). The objective of the dogs is to move all the sheep from a starting position to an objective position in a minimum of time. A two-phase strategy is used to first gather the sheep into a single group, and then to drive the flock towards the goal.

Furthermore, from an application point of view, many works propose collaborative WSN-UAV systems (Popescu et al., 2019). The vast majority of these works do not consider the problem as the coupling of two complex systems but as the creation of a heterogeneous multi-agent system (containing sensor agents and UAV agents).

The systems we deal with in this work have particular characteristics which constitute constraints to be taken into account when realizing the coupling. The first characteristic is the physical distribution of the system components, which leads to a lack of overview of the system at the agent level. This means that any form of centralization in the proposed approach must be excluded. Another constraint is that we consider the systems to be off-the-shelf. This leads us to consider agents as black boxes, i.e., an object that can be viewed only in terms of inputs and outputs without any modification of its code. Some solutions meet this constraint, such as the EAgilla (Lingaraj et al., 2018) whose deployment consists in introducing a middleware between the OS of the already deployed agent and the mobile agents. It allows a deployment without modifying the agents’ code, but imposes other constraints including a control of the agents’ operating system.

In contrast to other approaches, we will also attach great importance to non-functional requirements. This is due to the embedded nature of the systems we are interested in. Thus, we have to take into account the resource limitations (energy, memory, etc.) of the system components, as well as the criticality of the targeted applications which may require to respect reactivity, liveliness and timeliness (soft real-time). Note that solutions aiming at coupling a WSN and a system of UAVs generally take into account these limitations.

Our study of existing work has led us to deploy a set of techniques that we deemed appropriate to our context, given the satisfactory results they provide for the understanding and/or detection of collective product (David et al., 2009; O’Toole et al., 2014; Morris-Martin et al., 2019). These techniques are the use of probes, meta-knowledge and external observations. By classifying the proposed approaches according to their use of the three techniques (Table 1), we found that none of them exploits their complementarity.

**TABLE 1:** Work on emergent phenomena exploitation.

Work	Phases			Techniques			System constraints			
	Understanding	Detection	Exploitation	Probes	Meta-knowledge	External observations	Physical distribution	Inaccessibility	Resource limitation	Real time
Mataric. (1993)	•				•	•				
									
Dessalles and Phan. (2006)	•	•			•					
									
Klein et al. (2008)		•				•		•		
									
David et al. (2009)	•	•			•	•				
									
Lamarche-P et al. (2014b)	•	•		•						•
									
O’Toole et al. (2014)		•					•			•
									
Will. (2016)	•					•	•			•
									
Basso et al. (2016)	•	•				•				
									
Jones. (2010)	•	•				•	•		•	•
									
Serrano et al. (2009)	•					•		•		
									
Singh et al. (2017)	•	•				•				
									
Liu et al. (2018)	•	•		•		•				
									
Chen et al. (2008)	•	•		•		•				•

We also classify the approaches according to their consideration of non-functional constraints. We note (Table 1) that the proposed approaches do not take into account in particular the inaccessibility and the real-time aspect. Therefore, our approach will aim at filling these gaps.

## 3 The COPE Approach

This section presents the COPE (COllective Product Exploitation) approach which enables an embedded multiagent system to exploit the collective product of another one. The approach is based on the deployment of meta-agents in the both MASs. In the following, we call *producer system* the MAS from which emerges the collective product to exploit and the *consumer system* the MAS that will exploit this product. Naturally this has led us to distinguish two roles in meta-agents (and two sub-systems). The meta-agents deployed in the producer MAS are called producer meta-agents because their role is to essentially sense the collective product. The meta-agents deployed in the consumer MAS are called consumer meta-agents because their role is to exploit the information perceived by the producer meta-agents in order to exploit the collective product.

A meta-agent is a software/hardware agent that is external to the CPCs but is associated with one of their agents. It monitors the activity of the agent with which it is associated and can fake its perceptions to make it adopt a specific behavior. Since the agent is a black box, faking its perception represents a solution to make it react in favor of the implementation of the exploitation of the collective product without having to change its native behavior (which is usually not possible with off-the-shelf systems).

The deployment of meta-agents can be achieved in several ways:

• The meta-agents have their own dedicated hardware platform. This real autonomous embedded system can be glued to the chassis of the associated agent. Observing the agent can be done by listening the communication channel used by the agent’s communication interface. To act on the agent, it is necessary for example to send it messages.
• The agent’s hardware platform can be used to deploy the meta-agent. The associated agent is then a host agent. If a framework like OSGi is used, the meta-agent can then be deployed as an OSGi bundle. Observing the agent can be done by eavesdropping on the communications between the agent software components^3^. Acting on the agent can then be done by accessing the components deployed on the framework.

In any case, the system constituted by the meta-agents can be seen as a distributed and decentralized control system. In the illustrative scenario, we consider the most general case: the agents are considered as black boxes and the agents’ hardware platforms do not host the meta-agents.

The producer meta-agents provide indicators related to the collective product. The processing of these indicators allows the consumer meta-agents to act on the agents of the consumer MAS in order to adapt its global dynamics to exploit the collective product.

### 3.1 The Components of a Meta-Agent

The collective product emerges from agent interactions in the producer MAS. The meta-agents allow an external observation of the collective product in order to enable its exploitation. They use information on the agents, their interactions, their organization and their environment. The associated agent of a meta-agent can be viewed in terms of its message input and output, without any access of its internal functioning. A meta-agent then must eavesdrop agent messages to induce its state and its behavior.

**FIGURE 2:**
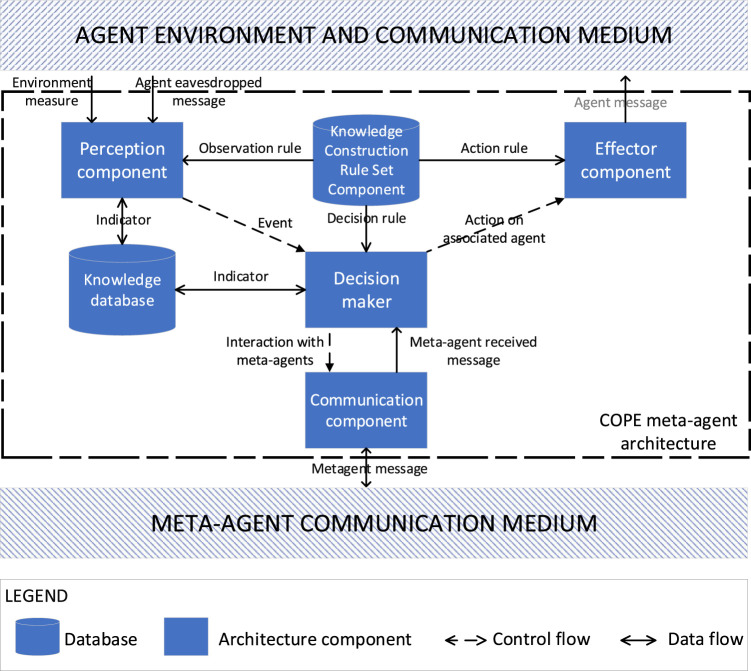
The components of a meta-agent.

The architecture of a meta-agent is built around six main components (see Figure 2).

The *Perception component (P)* eavesdrops the agent’s messages. According to the information encapsulated into the communication protocol messages specific to the producer and the consumer MAS, it is possible to extract information about the state of the agent, its role in the organization, its perception of the environment, etc. A meta-agent can enhance its representation of the environment with its own possible physical sensors.

The *Knowledge Construction Rules Set component* (*KCRSet*) is a set of rules for merging this measured information into indicators which are related to the different dimensions of the system (agent, interaction, etc.). For the producer meta-agents, these indicators are about the collective product occurrence and its exploitation by the consumer MAS. For the consumer meta-agents, the indicators are essentially linked to the integration of producer meta-agents indicators into the decision-making aspect of their associated agents so that they can exploit the collective product.

The *Knowledge database* (*KBase*) of a meta-agent includes knowledge about its environment, its own internal state and the states of its neighboring meta-agents. The internal state of the meta-agent includes plans that are currently being executed. The values of the indicators are also stored in this database.

The *Decision-maker component* (*DM*) is responsible for making decisions about the interactions to be carried out (with the associated agent or other meta-agents) to allow the exploitation of collective product. Producer meta-agents share the individually constructed indicators by interacting together. They ensure that they do not disrupt the behaviour of the producer MAS. They also interact with the consumer meta-agents to communicate consolidated indicators. A consumer meta-agent interacts with the producer meta-agents to request the indicators necessary for the exploitation of the collective product. It also interacts with consumer meta-agents to implement the exploitation of the collective product.

The *Effector component* (*E*) allows the meta-agent to act on the agent’s behavior by faking its perceptions through the generation of messages in the specific language of the MAS. The effector component of the producer meta-agent thus makes it possible to trigger specific behaviours in order to favour the exploitation of the collective product. The effector component of the consumer meta-agent modifies the perception of its associated agent to induce it to act according to its plan in order to achieve a specific goal related to the exploitation.

The *Communication component* (*C*) allows the cooperation of meta-agents by allowing interaction between them through the sending and receiving of messages.

### 3.2 Meta-Agent Activity

The activity of meta-agents is organized around three processes.

**FIGURE 3:**
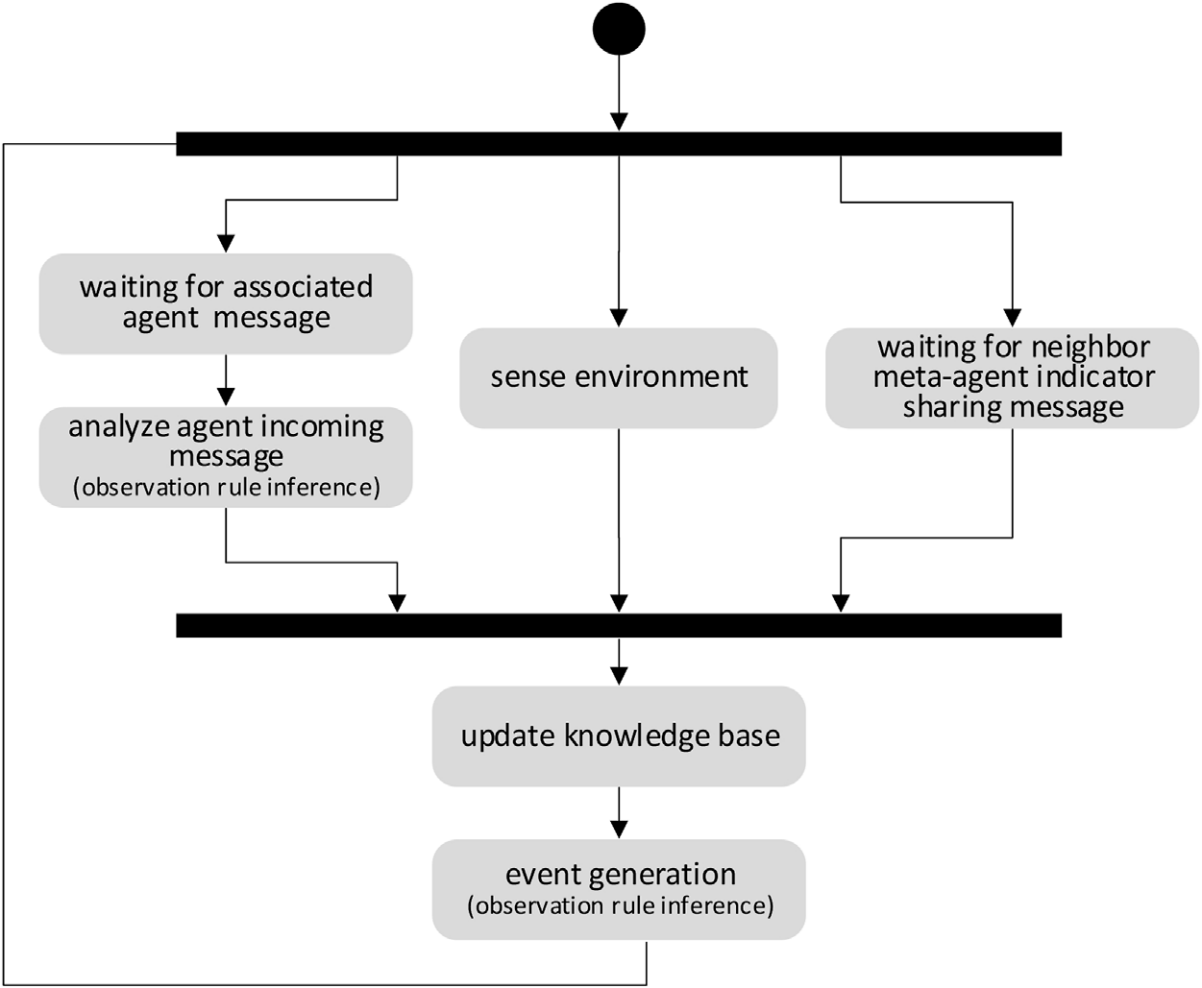
Updating the world representation of a meta-agent.

*Process 1—Updating world representation:* the objective of this process (Figure 3) is to ensure that the representation of a meta-agent of its environment is consistent with reality. Two tasks run concurrently for this purpose. The first one relies on using by a meta-agent of its own sensors or exploiting the measurements of its neighbour meta-agents. The second consists in exploiting the messages of the associated agent. The Perception component interprets listened messages by applying the KCRSet rules. The collected knowledge is stored in the KBase. It compares the new knowledge collected to the knowledge already stored in the KBase database in order to generate an event notifying the change of state to the decision maker component.

For example, let’s consider an application message sent by an agent who transmits a temperature to another agent. This message is listened by the meta-agent who will apply rules allowing it to i) understand that the message contains a measured temperature and ii) capture the temperature value. Another rule will allow to estimate the probability of the presence of a fire by following the temperature evolution using a temperature history stored in the KBase which lead to the generation of a “fire suspicion” event.

**FIGURE 4:**
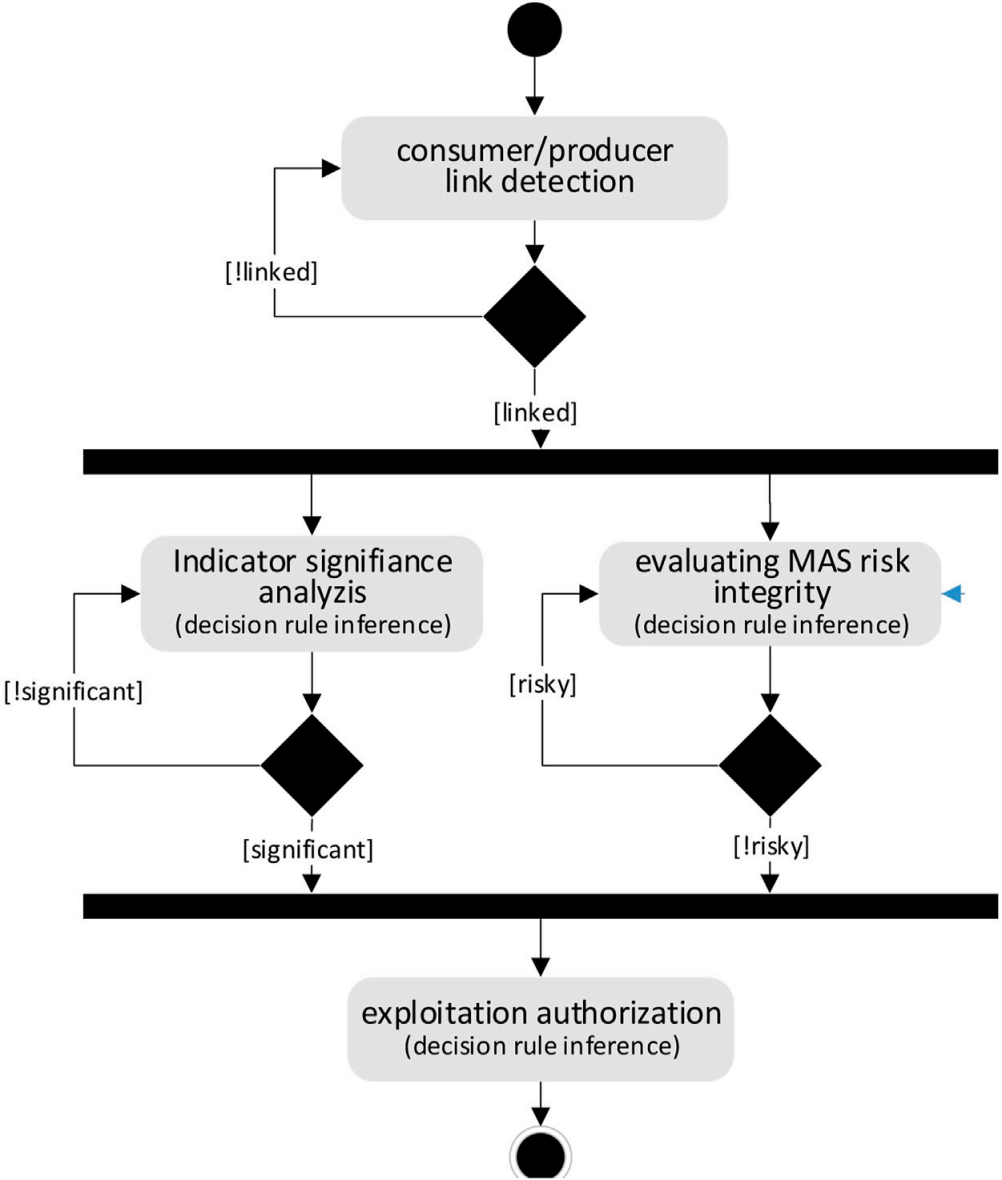
Authorizing the exploitation of the collective product.

*Process 2—Authorizing exploitation of collective product:* the objective of this process is to ensure that all the conditions^4^ have been met to allow the exploitation of the collective product (Figure 4). The first condition is the ability of the consumer meta-agents to communicate with the producer meta-agents. To this end, the consumer meta-agents send periodically discovery messages (*hello messages*) to announce their presence and inform that they are ready to use the collective product. The second condition is used to check that exploitation request of the collective product was issued, that the collective product indicators are significant and that using the product does not involve a risk to the integrity of the producer MAS. When a producer meta-agent detects a discovery message, it interacts with other producer meta-agents in order to know if the exploitation of the collective product can be operational. To make such decisions, the DM relies on rules contained in the KCRSet and on the knowledge stored in KBase. For example, in our scenario, if the indicators relating to the collective product of the MAS “sensor network” show that no route is yet found by the MWAC model, then the second condition is not satisfied. Producer meta-agents will respond unfavourably to the exploitation request.

**FIGURE 5:**
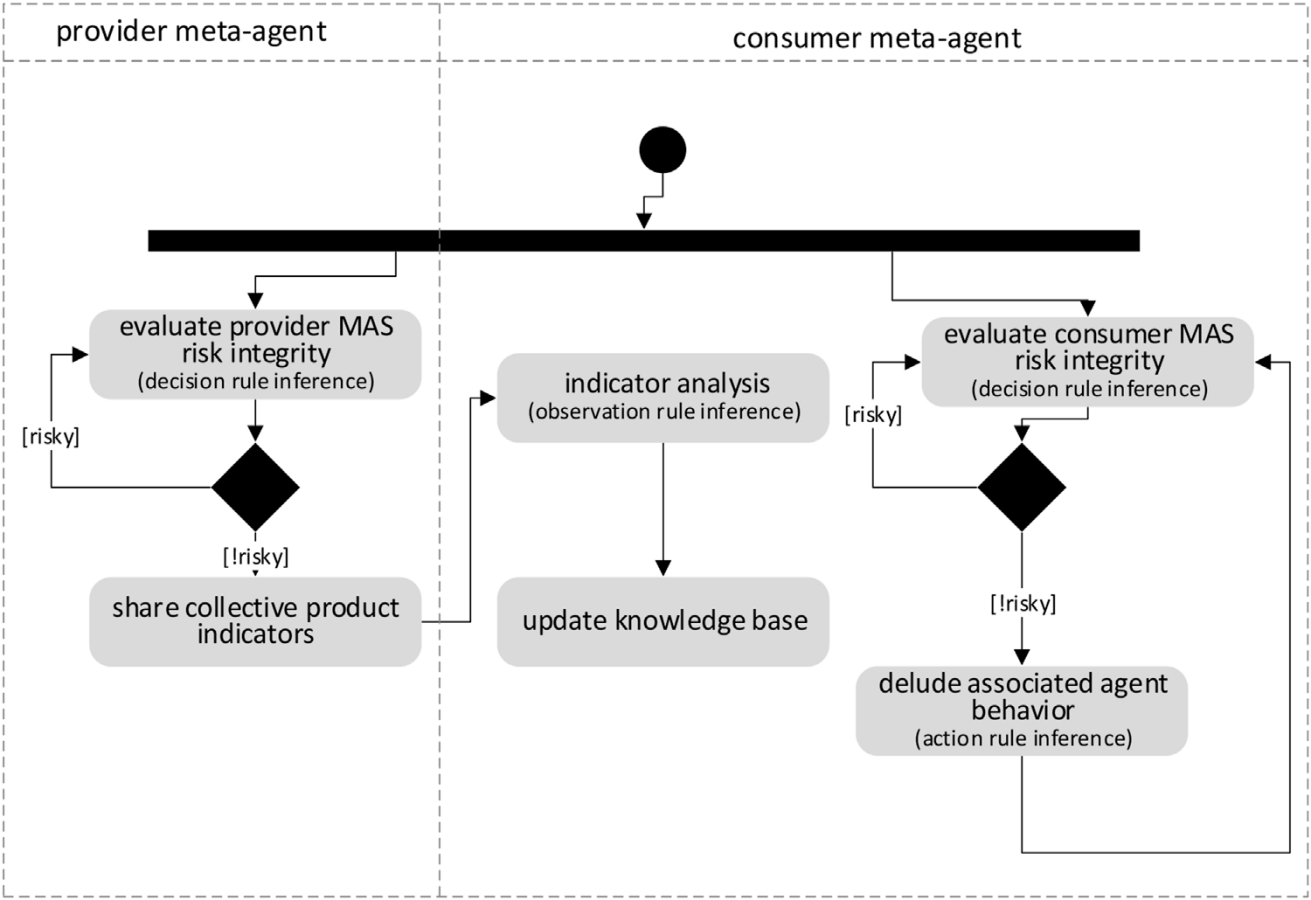
Exploitation of the collective product.

*Process 3—Implementing the exploitation:* the objective of this process is to enable consumer meta-agents to influence the behaviour of the consumer MAS through their agents (Figure 5). First of all, the producer meta-agents must allow the consumer meta-agents to follow the evolution of the indicators (according to an interaction protocol involving producer and consumer meta-agents). The producer meta-agents will fake the individual perceptions of the agents of the producer MAS by applying action rules of the KCRSet which aim to generate messages in the agents’ medium. During this process, the meta-agents of both systems shall also ensure that they do not negatively affect the operation of the two MASs. For example, in our scenario, when a MWAC route is exploitable to guide the movement of the UAVs (of the consumer MAS), a producer meta-agent associated with a sensor agent belonging to the route will periodically send out messages^5^ containing the expected indicators. These indicators will enable the UAVs to be oriented. Consumer meta-agents will share this information with each other. By applying rules of KCRSet, they will act on the behaviour of the UAVs in order to modify the movement of the UAV system.

### 3.3 Interaction Between Meta-Agents

**FIGURE 6:**
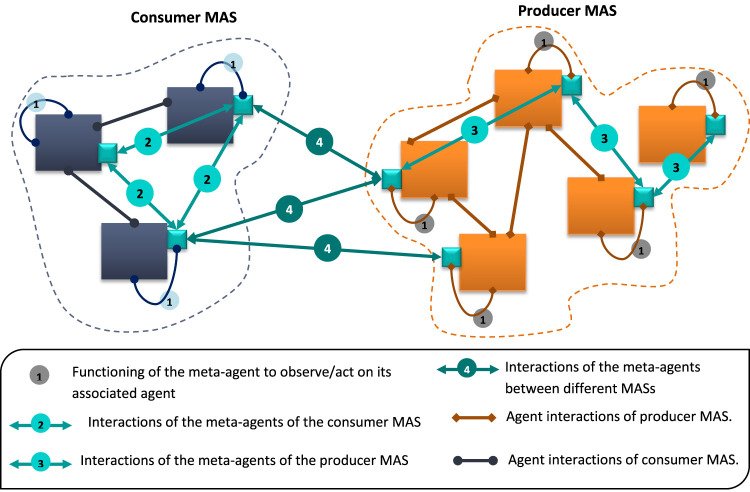
The different interactions of the meta-agents.

Meta-agents must interact to 1) determine whether collective product can be exploited and 2) to implement such an exploitation. For this purpose, several interactions are necessary (Figure 6). A meta-agent interacts with its associated agent to fake its perception and prompt it to respond as desired. It also interacts with meta-agents belonging to its own sub-system and to the other sub-system.

**FIGURE 7:**
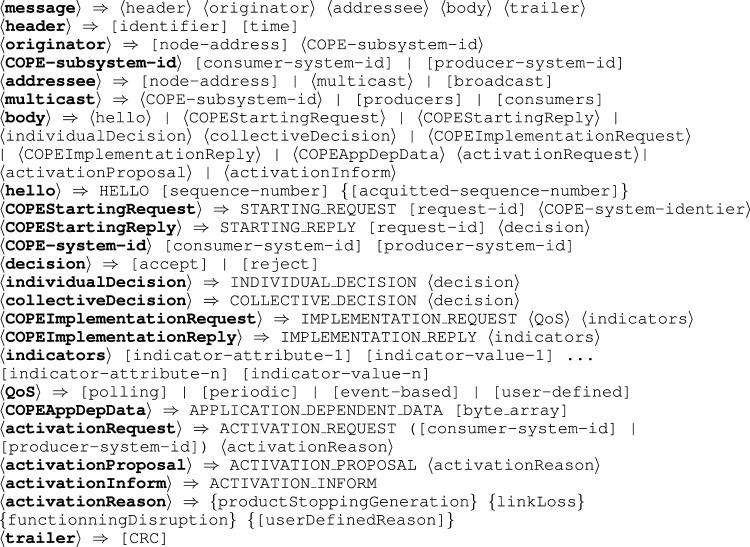
Message specification.

*Message format* The protocols we are going to present use messages specified according to the BNF notation in Figure 7. Non terminal symbols are enclosed by ⟨ ⟩ when expansion is detailed and by [ ] when the specific expansion is out of the scope of this discussion. Terminal symbols are not enclosed. Optional fields are enclosed by { }.

*Meta-agents Interaction protocol* All meta-agents periodically send hello messages to find out who their neighbors are. A neighborhood table contains for each neighbor, its network node address but also the unique COPE subsystem identifier (COPE-subsystem-id).

**FIGURE 8:**
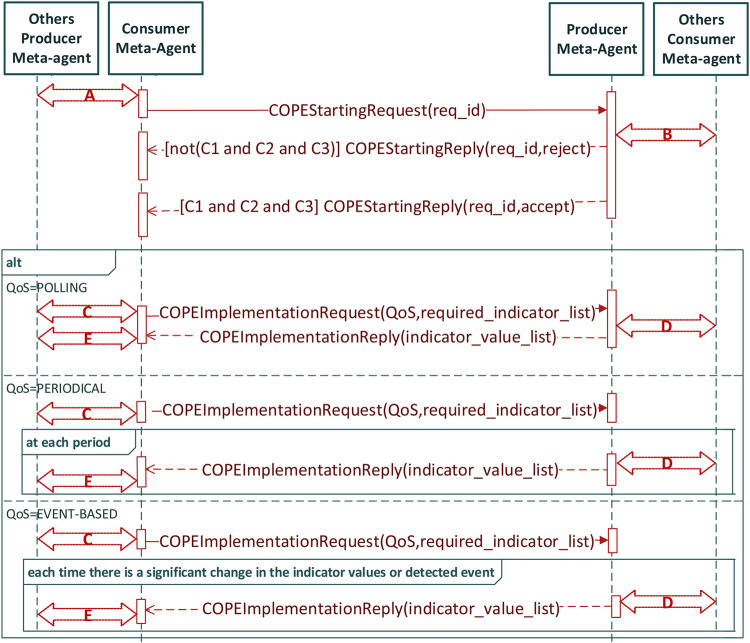
Interaction protocol focused on a producer meta-agent linked to a consumer meta-agent.

When a consumer meta-agent detects a producer meta-agent from the COPE subsystem it must cooperate with, it will generate the message COPEStartingMessage (see Figure 8). The sending of this message can be conditioned by the respect of an application-dependent protocol A (for example to charge the consumer meta-agents in connection with the producer meta-agents to send the request). The messages of these protocols are transported in messages COPEAppDepData.

Any producer meta-agent who receives a COPEStartingMessage verifies that it can authorize the exploitation of collective product (which may require the use of an application-dependent protocol B). Acceptance or possible rejection is notified *via* a COPEStartingReply message. In case of rejection, the consumer meta-agent applies a StartingReset procedure (by default a timer).

In case of acceptance, the consumer meta-agent will specify the list of indicators it wishes to obtain and demand a quality of service via a message COPEImplementationRequest. The establishment of this list may or may not require, depending on the application, interactions in the producer sub-system (application-dependent protocol C). Several QoS strategies are proposed (polling, periodical sending, event based) but they can be customized by the user.

The reception by a producer meta-agent of a COPEImplementationRequest can lead to the use of a specific protocol, in particular to adapt the behaviour of neighbouring producer meta-agents (application-dependent protocol D allowing, for example, producer meta-agents to send their own indicators in order to consolidate the information sent back). The values of the requested indicators will transit via the messages COPEImplementationReply.

The reception of a COPEImplementationReply message will generally lead to interactions specific to the consumer subsystem to, for example, share this information, or locally adapt the behaviour of the MAS (application-dependent protocol E).

*Non-functional constraints modulating the interaction* In embedded systems, many non-functional constraints have an increased importance in comparison to purely software systems. The need to save resources such as computing time, memory or energy leads us to introduce two states of a meta-agent: the “active” state and the “passive” state.

A meta-agent in the passive state only executes its individual processes: it keeps its KBase up to date. It does not actively participate in any interaction. It will exit this standby state if its activation rule is true or if it has received an activation request from one of its neighbors.

The activation rule takes into account several elements such as the role played by the associated agent in the generation of the collective product (C1), the capacity of the meta-agent to be reachable by meta-agents of the other subsystem (C2) and it capacity to operate without disturbing its associated agent (C3). Other application-dependent conditions can be defined by the COPE user.

To change the state of a meta-agent, the messages activationProposal and activationInform must be used.

## 4 Application

In this section, we introduce some elements of implementing COPE meta-agents in the application context we have presented.

### 4.1 Identification of Indicators

As soon as a sensor agent detects the fire, it generates a MWACRoutedData message to be sent to the LACU via the MWAC route. When a meta-agent of the consumer system detects a MWACRoutedData message, it can access the included network layer address to know the alert initiator and the datalink layer address to know which agent is the transmitter of the frame. Since the alert is sent out periodically by the sensor agents, if the consumer meta-agents bring their associated UAVs closer to this relay agent (by faking their perception to make them decrease the altitude), they will be able to pick up the message transmitted to it and thus discover the predecessor in the route. This predecessor can either be the creator of the alert or another relay agent closer to this creator.

### 4.2 Rules Definition

To parameterize the meta-agents, the designer has to define event driven rules respecting the following format:ON event IF condition THEN action

If the specified event occurs and an associated condition is fulfilled (at the time of the event occurrence) then the associated action is launched. The “event” part is generated by the perception component indicating that something significant to the system execution happens. The condition part is a logical test that, if true, will trigger the action.

**Updating world representation** To update its representation of the world, a meta-agent uses observation rules. These rules interpret the individual level behavior of agent to which the meta-agent is associated in order to extract the useful knowledge. In our case study, revealing events to build the world representation are the frames circulating on the agent communication medium. Actions consist in updating the world representation values.

For example, observation rule OR1 allows to observe the occurrence of the MWAC route from producer system side.

OR1 : ON RECEIVE agentMsg IF agentMsg.type == AgentMessage.MWACRoutedData AND AgentMessage.isAlertMessage(agentMsg.data) THEN KBase.MWACRoute = true

The observation rule OR2 enables to follow the role evolution of the associated agent:

OR2 : ON SEND agentMsg IF true THENKBase.agentRole = AgentMessage.getRole(agentMsg)

**Sharing indicators** The so-called decision rules specify how the indicators should be shared. These rules exploit the available knowledge on the representation of the world and conclude with a decision.

In our case study, after initializing the exploitation, the consumer meta-agents of the UAV system send a COPEImplementationRequest message to start the collective product exploitation. As soon as the decision-maker of a producer meta-agent receives this message, it applies the decision rule DR1 that uses knowledge concerning the role of the agent to select the action to be performed. The objective of the producer meta-agent is then to allow the localization of the previous relay (pr) sensor agent in the route (the one that it heard the MWACRoutedData message carrying the alert) and that we will call the *pr* agent. The consumer meta-agent then needs to receive the wireless physical signals of the *pr* in order to locate it by using a RSSI based algorithm. The producer meta-agent will therefore fake the perception of its associated sensor agent to transmit a MWACRoutedData message to *pr* with the objective that *pr* replies a message to it. It will be the effector component that creates the message when it has been notified of the decision by the decision-maker component.

DR1 : ON RECEIVE COPEImplementationRequest IF (KBase.agentRole == REPRESENTATIVE OR KBase.agentRole == LINK) THEN Decision.act(SEND, AgentMessage.MWAC RoutedData)

When a consumer meta-agent receives an indicator (i.e., a message from a producer meta-agent that allow to locate the previous relay), it applies the DR2 rule in order to move closer to the previous relay.

DR2 : ON RECEIVE COPEImplementationReply IF (KBase.AgentState == MOVING) THEN Decision.act (SEND, Agent Message.set Target,COPEImplementationReply.getNextPosition ())

**Faking agent’s perception** To fake the perception of its associated agent, the meta-agent uses action rules. As soon as the effector component of the meta-agent receives a decision from the decision-maker component, it infers the action rules contained in the KCRSet.

For example, rule AR1 guides the effector component to operationalize the decision to send a data message by luring the agent’s perception. The effector role is to embody this requested action by sending a MWACRoutedData message from the associated agent to the previous relay.

AR1 : ON NEW decision IF decision.equals (SEND, AgentMessage.MWAC RoutedData) THEN Effector.sendAgentMessage(Agent Message.MWACRoutedData, KBase.agentIdentifier, KBase.prevRelayIdentifier).

## 5 Evaluation of the COPE Approach

In this section, several simulations are presented to evaluate our proof-of-concept implementation of the COPE approach. We illustrate how COPE works by analyzing the network traffic. Then we verify that our approach meets the need to allow UAVs to move to the fire area. To do so, we measure the travel time of the UAVs from the LACU to the fire. Finally, we discuss the cost of using COPE.

Experiments were performed with the Multi-Agent Software Hardware simulator (MASH) (Jamont et al., 2013; Jamont and Occello, 2015), dedicated to the design and deployment of cyber physical systems.

In order to have a comparative study, COPE is compared for these criteria to a state of the art solution named EAgilla (Lingaraj et al., 2018). EAgilla is a middleware specialized in the development of applications using information coming from a WSN. EAgilla is not designed to exploit collective products but its usage scenario shares some properties with our scenario.

### 5.1 Illustration of How COPE Works

**FIGURE 9:**
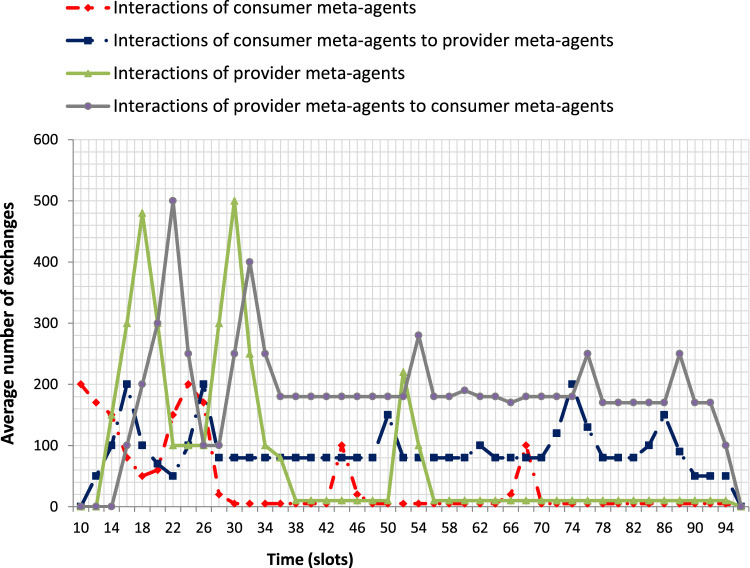
Meta-agents interactions of producer and consumer MASs.

**FIGURE 10:**
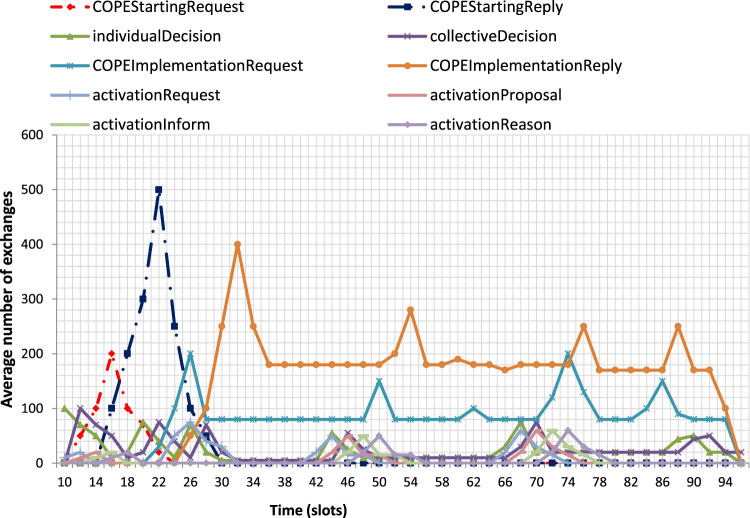
Evolution of the different types of meta-agent interactions.

To illustrate the functioning of our solution, we use a WSN of 100 nodes and we put ourselves in the conditions of a swarm of 50 UAVs (we title this configuration by Config 1). A first set of measurements shows the number of interactions involving meta-agents (Figure 9). A second set of measurements shows the type of messages exchanged (Figure 10). We can thus distinguish 3 phases:

*Authorization of the exploitation:* From *t* = 6 to *t* = 18 slots we note a large number of message exchanges between producer meta-agents that correspond to the decision to authorize the exploitation of the collective product. We note an even larger number of interactions between the consumer meta-agents which leads to the decision to exploit the collective product of the other system. We notice that the interactions between the consumer meta-agents and those with the producer meta-agents precede the exchanges between producer meta-agents. Indeed, the consumer meta-agents must have expressed their need to exploit the collective product before the other meta-agents work on collecting indicators.

By analyzing the graph of Figure 10 on the same interval, we can see that the messages exchanged during this phase are *COPEStartingRequest* sent by the consumer meta-agents to the producers, followed by *COPEStartingReply* messages generated by the producer meta-agents to the consumers. At *t= 14 slots* we notice a peak of *COPEStartingRequest* messages which reflects the involvement of a large number of consumer meta-agents in the process in order to be able to share the information with as many producer meta-agents as possible. It is for a similar reason that we see a peak of *COPEStartingReply* messages at *t= 16 slots*. Interactions between meta-agents in the same system (consumer or producer) induces *individualDecision* messages and *collectiveDecision* messages as well as messages of the meta-agent state adaptation mechanism (*activationRequest, activationProposal, activationReason, activationInform*).

*Implementation of the exploitation:* From *t* = 19 to *t* = 90 slots we notice that the meta-agents of both systems interact throughout this interval to allow the exchange of indicators. At the beginning of the period and a few times during this interval (for example at *t* = 50 slots), traffic peaks of the producer meta-agents represent the taking of collective decisions authorizing the transmission of indicators required by the consumer system. The interactions between the consumer meta-agents are present at the beginning of this interval and reappear at *t = 42 slots* and at *t = 66 slots* to guarantee the successful transmission of the exploitation requests.

By analyzing the graph of Figure 10, we note that the messages exchanged are *COPEImplementationRequest* (consumer meta-agents) and *COPEImplementationReply* (producer meta-agents). At *t= 44 slots* the interactions between consumer meta-agents show in particular the involvement of new meta-agents in the interactions allowing the exploitation (messages *activationRequest, activationProposal, activationReason, activationInform*). This increase in the number of participants is naturally followed by an increase in the messages *COPEImplementationRequest* at *t= 50 slots* which will imply an increase in the producer meta-agents involved (activation message) at *t= 52 slots* and finally the increase in messages *COPEImplementationReply* at *t= 54 slots*.

*Arrival of the UAVs on the fire zone:* from *t* = 91 to *t* = 96 slots the interactions of the consumer meta-agents towards the producers start to decrease which implies the decrease of the interactions of the producer meta-agents towards the consumers. The consumer meta-agents progressively reach their objective which leads them to stop sending *COPEImplementationRequest* messages. We notice then that the producer meta-agents are less solicited by observing the decrease of response sending (messages *COPEImplementationReply*).

*About implemented coupling strategy:* the establishment of a coupling strategy requires a compromise between several criteria, including the simplicity of the definition of the rules to be implemented in the meta-agents and the QoS criteria. The strategy we proposed to address the problem of the illustrative scenario is not optimal in terms of energy consumption^6^ because the identified route may not be the optimal route to be followed by the UAVs. However, it better illustrates the coupling of two physically distributed systems following a decentralized approach.

### 5.2 Ends-To-End Delay

**FIGURE 11:**
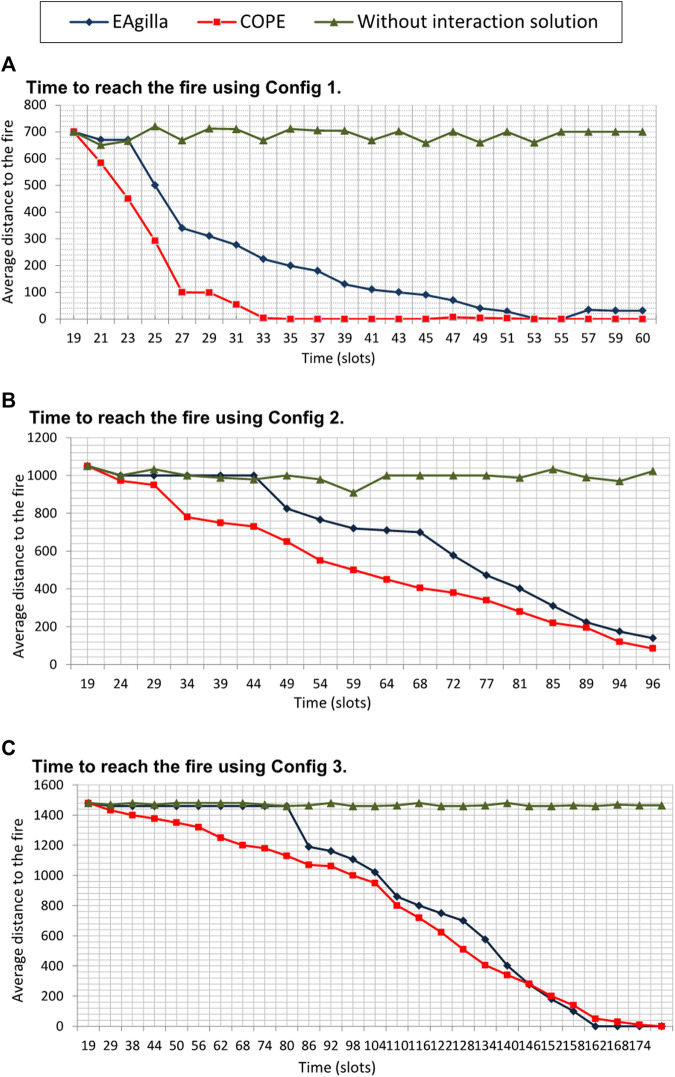
Time to reach the fire by the UAVS.

We test whether the COPE approach allows UAVs to reach the fire as well as the influence of system density on its operation using three configurations where density of WSN nodes is decreased (density of sensors divided by 2 for Config 2 then 4 for Config 3). By analyzing the obtained results (Figure 11), we notice that with COPE the UAVs start to approach the fire faster than in the implementation using EAgilla. Following the EAgilla approach, mobile agents traverse the network in search of a shorter path and do not exploit the available collective product. The additional time required by EAgilla (23 slots in Figure 11A, 44 slots in Figure 11B, 80 slots in Figure 11C) is due to the time required for agent cloning, agent mobility (of their code and data vector) and agent coordination.

We noticed that COPE becomes slower when the MAS density decreases because of the communication link loss. More time is then needed to execute the meta-agent activation mechanism. For Config 1 and Config 2 we find that COPE is faster than EAgilla, and that both solutions give almost the same result when the network density is less important with Config 3 (Figure 11C).

However, it should be noted that in this simulation, we consider that the alerts are sent continuously. The further apart in time they are, the longer it takes for UAVs to progress along the MWAC route.

**FIGURE 12:**
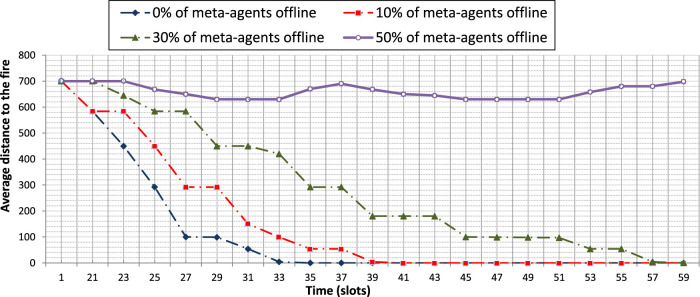
The influence of the meta-agent density on the time needed for the UAVS to reach the fire.

We evaluate the influence of the density of the meta-agents on the performance of our approach. To this end, we use Config 1 where we will disable a set of randomly chosen meta-agents. We set the proportion of disabled agents at 10, 30 and 50% of the total number of deployed producer meta-agents. The results obtained on the graph of Figure 12 show that when 10% of the meta-agents are disabled the UAVs move more slowly towards the fire. This is explained by the time needed (e.g., between *t = 27 slots* and *t= 29 slots*) to engage more producer meta-agents in the interactions in order to meet the demands of the consumer meta-agents to share the indicators needed for the collective product exploitation. Indeed, the meta-agents have some mechanisms (e.g., timers) to decide to start interacting in order to change the state of some standby agents to the active state.

For the same reason, with 30% of the meta-agents disabled, we notice that the UAVs move even more slowly towards the fire. (Figure 12). The activation mechanism is triggered more frequently (e.g., at *t= 25 slots*, *t = 29 slots*, *t = 39 slots*…).

With 50% of meta-agents disabled, the route is not always perceived by the consumer meta-agent. So, their associated UAVs must explore the environment more widely to fill this information gap. More complex rules for efficient exploration of the environment should be defined. These rules will also help to break the one-agent/one-meta-agent association.

### 5.3 COPE Cost

**FIGURE 13:**
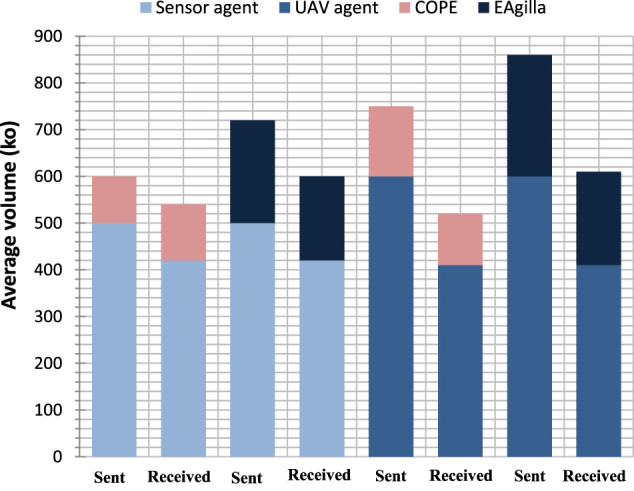
Cost of COPE vs. EAGILLA.

The components of the two systems are limited in resources, especially energy, which defines their lifetime. We test the cost of the approach in relation to the rate of exchanged messages because the communication module is the one that consumes the most energy of the components of the two systems. The obtained results (Figure 13) by using Config 1 show that COPE adds 15% more exchanges to the average exchange volume of already deployed agents because the operation of the solution relies on collective decisions involving several meta-agents interactions. EAgilla adds 20% due to the mobility of the agents, which is done by sending/receiving messages.

Concerning the sensor agents which are involved in the first four bars on the graph of Figure 13, we notice that our approach (the first two bars) adds a similar volume of sent and received messages because each meta-agent which receives an individual or collective decision makes its own decision and sends it back to the meta-agents in its neighborhood only.

For the EAgilla middleware, we also see that the volume of messages sent and received by the sensor agents are almost equal because these communications represent the mobility of the mobile agents of the middleware in order to find the shortest route to the LACU (each message containing the code of a mobile agent received is systematically sent afterwards).

Regarding the UAV agents that are involved in the last four bars of the graph, we note that the COPE approach adds an approximately similar volume of sent and received messages, but this volume remains relatively high compared to that of the sensor agents. This can be explained by the fact that the meta-gents associated with the UAVs are consumers, so they exchange more frequently with each other and with the producer meta-agents associated with the sensor agents in order to benefit from the collective product of the producer system.

For the EAgilla middleware we also notice that the volume of messages sent and received by the UAVs agents is even more important than the volume of the messages of the sensor agents which is due to the movement of the mobile agents in order to guide the swarm by using an RSSI based algorithm and the tuple space of the middleware.

In the test we performed, COPE appeared to be less expensive than EAgilla.

## 6 Conclusion and Future Work

In this paper, we proposed an approach called COPE (COllective Product Exploitation) to allows a MCPC to exploit the collective product of another one. This approach relies on the use of meta-agents deployed in the two systems to be coupled. The solution we propose here can be characterized as follows:

• Non-intrusive: by using the ability of meta-agents to observe the occurrence of a collective product and to make decisions in order to allow its exploitation without any cooperation from the MCPC agents. No modification of the decision loop of these agents is indeed necessary.
• Generic: the components of a meta-agent are generic and must be parameterized via the definition of perception, decision and action rules.

We are presently working on improving the following characteristics of the proposed solution:

• Multi-dimensional compliance: by allowing the exploitation of several MASs with several collective products. We need to validate the operation of the model with multiple producer MASs and a single consumer MAS initially, and then generalize to multiple consumer MASs. An interesting problem will be the study of the impact of negative interference.
• Environment interaction awareness: by not only taking into account the eavesdropping of the exchanged frames, but also all the possible interactions *via* the environment between a meta-agent and its associated agent. This means that the meta-agents must be able to perform an activity recognition of their associated agents.
• “One-agent/one-meta-agent association” breaking: by associating one meta-agent to a set of agents. To do this, a meta-agent must be able to observe all the agents in its communication range and to influence them to realize the exploitation of the collective product. An issue lies in identifying these different agents. The loss of a communication link between producer and consumer meta-agents could no longer be considered as a disturbance but as a normal functioning.

We also intend to implement the model in the context of Web Of Things (WoT) applications considering a Smart Home application (Khenifar et al., 2014). In a such scenario, a home security system and a home comfort management system are studied. Each one is incorporated in an independent home. The objective is to enable the exploitation of these systems by using COPE with a RESTful implementation.

## Data Availability

The original contributions presented in the study are included in the article/Supplementary Materials, further inquiries can be directed to the corresponding author.

## Footnotes

1 We do not go into the details of MWAC’s adaptation mechanisms to WSN topology changes and route QoS (congestion, error rate…) which may imply that the route that is actually followed by the message differs from the calculated route.

2 In order to simplify the technical explanations of how MWAC works and the COPE evaluation, the WSN is considered in this article without mobility.

3 In this case the agent is a “grey” box rather than a “black” box.

4 Possible application-dependent conditions should also be considered here.

5 Another possible implementation of this solution would have been to send an application-specific message.

6 From an energy consumption point of view, a better solution would be for a single UAV to locate the burning area using the identified route and transmit the location (e.g., a GPS position that it measures with its own sensor) to the other UAVs. Allowing this communication could force the UAV to return to the transmission area of the others.
